# Systematic Review on the Role of Lasers in Endodontic Therapy: Valuable Adjunct Treatment?

**DOI:** 10.3390/dj8030063

**Published:** 2020-07-01

**Authors:** Eugenia Anagnostaki, Valina Mylona, Steven Parker, Edward Lynch, Martin Grootveld

**Affiliations:** 1 Leicester School of Pharmacy, De Montfort University, Gateway House, Leicester LE1 9BH, UK; vasiliki.mylona@my365.dmu.ac.uk (V.M.); steven.parker@my365.dmu.ac.uk (S.P.); edward.lynch@hotmail.com (E.L.); mgrootveld@dmu.ac.uk (M.G.); 2 School of Dental Medicine, University of Nevada Las Vegas, Las Vegas, NV 89154, USA

**Keywords:** aPDT, endodontic therapy, laser, PBM, postoperative endodontic pain, root canal treatment, systematic review

## Abstract

(1) Background: Adjunctive instruments, such as lasers have been investigated to address the risk of failure of endodontic therapy due to the complexity of the root canal system. Lasers have been used therapeutically, in direct irradiation of the root canals or adjunct to irrigants placed into the canals, in combination with a photosensitizer (antimicrobial photodynamic therapy) and in pain management (photobiomodulation). The purpose of this systematic review was to evaluate the evidence in clinical use within these three areas of therapy. (2) Methods: PubMed, Cochrane and Scopus search engines were used to identify human clinical trials regarding the use of lasers in endodontic therapy. (3) Results: After applying the keywords and additional filters, inclusion and exclusion criteria, the initial number of 1486 articles was reduced to 17. It was revealed that almost all studies (14/17) presented a statistically significant improved outcome in laser-assisted endodontic therapy, with the remaining three not showing any adverse effects. (4) Conclusions: The use of laser photonic energy of appropriate delivered parameters can be proposed as useful adjunctive when considering optimal treatment modalities in orthograde endodontics. Additionally, a tendency of research towards pain modulation in this field is developing.

## 1. Introduction

### 1.1. Pulpitis—Apical Periodontitis

In deep carious lesions where microorganisms have invaded the dental pulp, a substantial inflammation known as pulpitis may take place. Usually, the inflammatory reaction remains localized even after the microorganisms have invaded the pulp cavity. Notably, as long as the pulp tissue is vital only a limited number of microorganisms reside inside the root canal, and hence the infection does not diffuse into root dentine. In this case, the treatment of choice is endodontic therapy, and its prognosis is excellent as far as microorganisms are concerned.

However, as a persisting infection it can potentiate the development of apical periodontitis, which is an inflammatory process of the peri-radicular tissues caused by microorganisms inside the root canal system. In apical periodontitis, the lesion contains phagocytes and other defense cells, which can, in turn prevent further proliferation of the microbial infection [1].

### 1.2. Endodontic Therapy

The goal of endodontic therapy (also known as endodontic treatment, or root canal therapy, or root canal treatment) is the elimination of diseased dental pulp tissue residue and the prevention of inflammation of the periapical tissue, or the control and elimination of microbial infection with the further aim of promoting healing in the case of persistent lesions [1].

The accumulation and persistence of microorganisms inside a necrotic root canal depends on the availability of oxygen and nutrients, along with the host’s immune defense; this leads to differential microfloral compositions. Despite the consequent variants of micro-organisms, the dominant species are anaerobes. Specifically, the proportion of the anaerobic microbial load is reported to lie between 70 and 100%. The main pathogen found in the event of persistent periapical infection is *Enterococcus faecalis*, which in view of its ability to form complex biofilms, and to survive without nutrients for months, belongs to one of the most resistant species [2]. The penetration of microorganisms into the surrounding dentine has been shown to occur via the dentinal tubules and contamination can reach a depth of approximately 1000 μm [3].

In order to combat the microbial challenge, the use of local antimicrobial irrigating solutions with tissue-dissolving ability, such as sodium hypochlorite (NaOCl), combined with mechanical instrumentation has represented the “gold standard” in endodontic therapy [4]. The penetration of NaOCl into root dentine is estimated to be ca. 130 μm [5]. Additionally, the complex three-dimensional anatomy of root canals has a limiting effect on the penetration of irrigants within this multiplex system. 

According to Haapasalo et al. [6], the successful elimination of endodontic infection depends on the following factors: (i) host defense system; (ii) systemic antibiotic therapy in some cases; (iii) chemo-mechanical preparation and irrigation; (iv) local root canal disinfecting medicaments; (v) permanent root canal obturation; and (vi) permanent restoration to achieve an effective coronal seal.

Systemic antibiotics are not predictable outcome treatments and should only be prescribed in cases of spreading infection and compromised hosts [6]. 

Unfortunately, a risk of failure has been described in the scientific literature of between 7 and 16%, and mainly through the complexity of the root canal system [7]. Following such failure, very often a re-treatment has to be performed and in this case the respective reported rate varies between 11 and 24% [8]. Hence, additional methods have been investigated to improve the cleansing and disinfecting action of irrigant solutions. 

### 1.3. Lasers in Endodontic Therapy

Apart from ultrasonic irrigation techniques, laser use in endodontics has been a major field of research since their introduction in dentistry. The interaction of near-infrared (NIR) wavelengths (810–1064 nm) with host tissue is predominately through a photothermal effect. In view of their high penetration depth into dental tissue, their penetration into dentine is shown to reach >1000 μm through scattering and transmission along the dentinal tubules, which in turn act as “light guides” [9]. Since NIR-laser photonic energy can be absorbed by chromophores such as melanin, only pigmented microorganisms will be susceptible to direct inactivation [10]. Additionally, an indirect microbicidal effect will occur from photothermal damage [11]. In vitro studies have been performed both in wet and dry root canals, with promising antimicrobial results. However, overheating and melting of the root canal walls is one drawback of laser use in dry environments [12].

In view of their strong broad absorption band from O-H bond stretching and inter-molecular associations through hydrogen (H)-bonding, lasers available in dentistry which operate within the mid-infrared region (2780–2940 nm, equivalent wavenumbers 3597–3401 cm^−1^), have been investigated more recently, not least because of their ability to cause a “cavitation” effect in such media. This depends on a rapid fluid motion in the root canal arising from expansion and implosion of bubbles at the laser tip, which are caused by the high peak power experienced during pulsed emission along with the high absorption in irrigating solutions. During laser-activated irrigation (LAI), as well as PIPS^TM^ (Photon Induced Photoacoustic Streaming) and SWEEPS^TM^ (Shock Wave Enhanced Emission Photoacoustic Streaming) techniques which rely on the same phenomenon, the movement of the irrigant is extremely turbulent, leading to improved removal of microorganisms and their biofilms from the root canal system. This action is based on a merely physical effect [4]. In addition, a chemical effect also appears to play a significant role, with an increased reaction rate of NaOCl upon activation by erbium lasers [13].

Laser use in endodontic therapy has further developed through antimicrobial photodynamic therapy (aPDT) and has been adopted as an adjunct alternative. This technique is based on a photosensitizer (PS) that is applied inside the root canal and after a particular incubation time, is irradiated by a light source, the wavelength of which coincides with the absorption band maximum of the photosensitizer. In the presence of oxygen, a reaction takes place which leads to the production of reactive oxygen species (ROS) and singlet oxygen (^1^O_2_), a process leading to microbial cell damage [14]. The benefits of this technique are the wide-spectrum of affected microorganisms, the absence of photo-resistant species after multiple applications and minimal damage to host tissue. Moreover, the treatment outcome is independent of the antibiotic-resistant pattern of the micro-organisms. The selectivity of this approach is dependent on the incubation time: microorganisms require minutes of exposure, but host cells require hours [15]. Nevertheless, inside the infected root canal system, the only existing cells are microorganisms. Most research has been performed with methylene blue (MB), toluidine blue (TBO), and indocyanine green (ICG) as photosensitizers and lasers as light sources with corresponding wavelengths of 660, 635 and 810 nm, respectively.

### 1.4. Post-operative-endodontic Pain

Except for microorganism infection, another major concern of endodontic treatment is the post-operative-endodontic pain (POP) experienced by a high number of patients. This has been described in the scientific literature as having a prevalence of between 3 and 58%, where the range in these reports can be explained by the use of differential criteria to assess POP [16]. When pain occurs after endodontic treatment, patients may consider the treatment per se as the causative factor and may question the clinician’s skills. Hence, the management of pain is of critical importance. 

The actual cause of post-operative-endodontic pain is considered to be an irritation of periradicular tissues associated with microorganisms, or a mechanically- or chemically-induced injury to the radicular area. Specifically, apical extrusion of tooth debris or irrigants, intra-canal dressings and micro-organisms might occur, resulting in inflammation and pain [17]. This irritation contributes to nociceptor activation and local inflammation, leading to a release of prostaglandins, bradykinin, leukotrienes, serotonin and cytokines from the injured tissues, which therefore potentiates peripheral sensitization [18,19].

Typically, the duration of pain lasts between 24 and 48 hours, with reports of pain persisting for three days following root canal treatment [20]. The suggested management of POP includes administration of non-steroidal anti-inflammatory drugs, paracetamol or corticosteroids. Recent studies have proposed pain management to be successful with photobiomodulation therapy (PBMT) [21,22,23]. 

### 1.5. Photobiomodulation (PBM)

PBM therapy (PBMT), through the application of photonic energy at specific wavelengths within the optical window of 650–1350 nm, works on the principle of inducing a biological response through energy transfer. Such non-ablative photonic energy delivered into tissues modulates biological processes within that tissue, and also within the biological system of which that tissue is a component part. In this context, cellular metabolism can be modulated, leading to secondary effects which modify cellular behavior [24]. The benefits of this approach can be described as anti-inflammatory, analgesic and therapeutic and with a correct incident dose applied, PBM therapy has no appreciable thermal effects in irradiated tissue [25].

In vivo studies have shown that PBM can inhibit nerve function. Other alterations include local conduction blockage, disruption of axonal flow, and specific nociceptor inhibition. All these changes give rise to pain relief and are reversible without side effects [23].

### 1.6. Aims of the Study

In this systematic review of randomized controlled clinical trials on lasers used as an adjunct in non-surgical endodontic treatment, three major fields have been explored:

1. Conventional laser use inside the root canal as an additional disinfection method;
2. Lasers combined with a photosensitizer inside the root canal in antimicrobial photodynamic therapy (aPDT);
3. Lasers in post-operative-endodontic pain management, coupled with photobiomodulation therapy (PBMT).

The aim of this systematic review was to evaluate which field is most strongly supported by clinical evidence, and if so, which shows more favorable results than application of the gold standard (endodontic) treatment alone.

## 2. Materials and Methods

### 2.1. Search Strategy

The search engines PubMed, Cochrane and Scopus were used with following keywords and combinations:

- (Endodontic OR root canal) AND (laser);
- (Endodontic OR root canal) AND (photobiomodualtion OR PBM OR LLLT OR photodynamic OR PAD OR photoactivated);
- (Endodontic OR root canal) AND (diode OR Nd:YAG OR erbium OR Er:YAG OR Er, Cr:YSGG)

After applying the additional filters (Clinical Trial [ptyp] AND “last 10 years” [PDat] AND Humans [Mesh] AND English [lang]), the initial number of 1486 articles was reduced to 66.

Titles and abstracts of the above articles were independently screened by two reviewers, and in the case of disagreements, this was resolved by discussion. The following inclusion/exclusion criteria were applied:

Inclusion criteria:

- Only randomized controlled clinical trials;
- Laser employed as an adjunctive therapy;
- Identical conventional endodontic treatment performed to all groups;
- Negative control group;
- At least 10 participants per group;
- In case of aPDT applied, correct combination of photosensitizer (PS) and laser used.

Exclusion criteria:

- No endodontic treatment applied;
- Apical surgery;
- Duplicates or studies with the same ethical approval number;
- No negative control group;
- Different conventional endodontic treatment applied to the test group;
- Low sample size (less than 10 per group);
- No randomized controlled clinical trials, case series or pilot studies;
- In vitro studies;
- LED used as light source.

After screening and implementation of the eligibility criteria, 17 articles were included in total, which were categorized in terms of:

- Conventional laser-assisted endodontic treatment (4 articles);
- aPDT in endodontics (5 articles);
- PBM in endodontics (8 articles)

**Figure 1:**
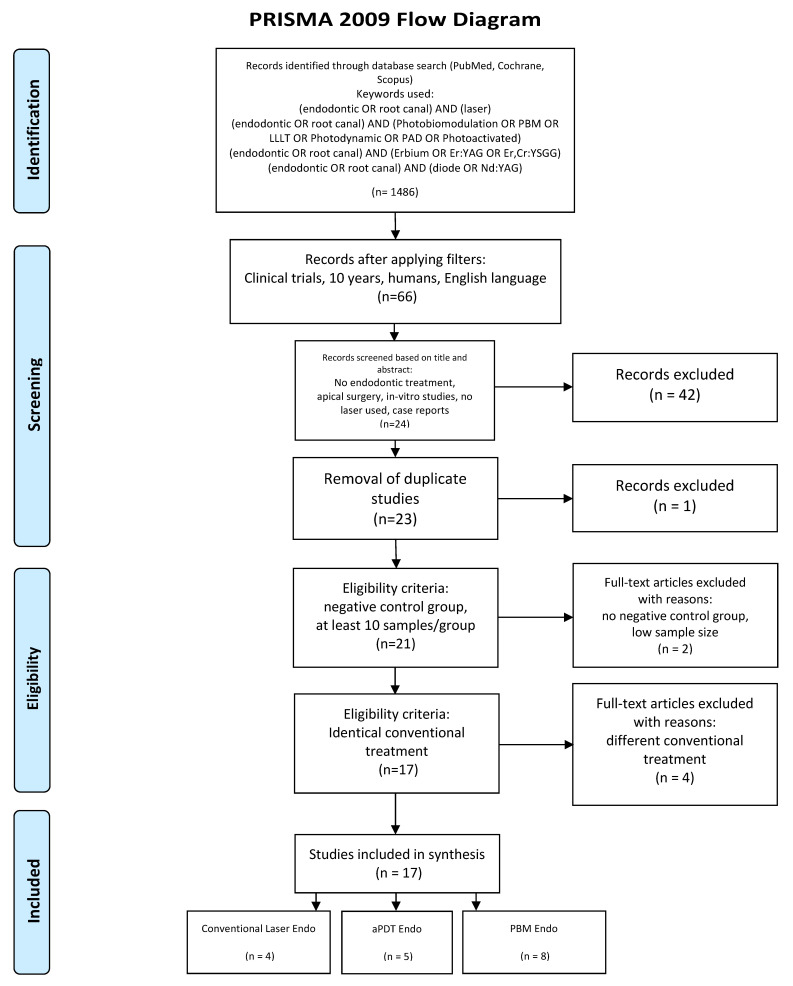
PRISMA flow-chart of selected criteria for the included article reports. (From: Moher, D.; Liberati, A.; Tetzlaff, J.; Altman, D.G.; Group, T.P. Preferred Reporting Items for Systematic Reviews and Meta-Analyses: The PRISMA Statement (Reprinted from Annals of Internal Medicine). *PLOS Med*. **2009**, *6*, e1000097, doi:10.1371/journal.pmed.1000097. [26]).

The search was performed from April 08 to April 15, 2020. The following flow-chart (Figure 1) which was prepared in accordance with the PRISMA guidelines [26], indicates the study selection process.

### 2.2. Data Extraction

Data extraction of the selected studies was based on the following factors:

- Citation (first author and publication year);
- Type of study/number of sampling participants;
- Test/control group;
- Aim/approach;
- Laser/protocol;
- Follow-up;
- Outcome.

### 2.3. Quality Assessment

Furthermore, studies were analyzed through a risk of bias assessment. The Cochrane Risk of Bias tool [27] was modified according to the requirements of this systematic review. 

The risk of bias was determined according to the number of "yes” or "no” answers to the following questions allocated to each study:

- Randomization?
- Sample size calculation and required sample number included?
- Allocation ratio of 1:1?
- Baseline situation similar?
- Blinding?
- Parameters of laser use described appropriately, and calculations correct?
- Power meter used?
- Numerical results available (statistics)?
- Outcome data complete?
- Correct interpretation of data?

The classification was performed according to the total number of “yes” answers to the above questions. The degree of bias was calculated as follows:

- High risk: 0–4
- Moderate risk: 5–7
- Low risk: 8–10.

## 3. Results

### 3.1. Primary Outcome

The aim of this systematic review was to evaluate the outcome of the studies and detect and analyze the missing parameters of their protocols. 

### 3.2. Data Presentation

The extrapolated data of each laser application category are presented in Table 1, Table 2 and Table 3.

### 3.3. Quality Assessment Presentation

The risk of bias of the included studies is presented in Table 4.

In total, 12/17 articles (70.5%) showed a low risk of bias, with one article [37] scoring 10/10, five [28,32,33,38,43] scoring 9/10, and six [30,31,34,35,40,42] scoring 8/10.

However, 5/17 articles (29.5%) showed a moderate risk of bias with three articles [29,36,39] scoring 7/10, and two [41,44] scoring 6/10.

The most common negative answers concerned the questions (a) use of a power-meter, (b) sample size calculation and required sample number included, and (c) correct description of the protocol.

The mean±standard error (SEM) score value was 8.46 ± 0.22.

### 3.4. Analysis of Data

Regarding the treatment outcomes, 14/17 articles (82.3%) presented a positive therapeutic result, with significant differences observed between the laser treatment and their respective control group, whilst 3/17 articles (17.7%) showed no significant differences between these classifications.

Specifically, for each laser application category, the studies with positive results were allocated as:

- Three of four studies [28,30,31] in conventional laser endodontic treatment;
- Four of five studies [32,33,34,35,36] in aPDT in endodontics;
- Seven of eight studies [37,38,39,40,41,42,43] in PBM in endodontics.

From these studies, 11/14 showed only a low risk of bias, whilst 3/14 showed a moderate risk.

Concerning the investigational objectives of the included studies, they were assigned as:

- Pain: [28,29,30,31,32,33,37,38,39,40,41,42,43,44];
- Microorganisms: [30,33,34,35,36];
- Radiographic healing: [33].

Hence, it is clear that the dominant research area was pain evaluation (14/17). Moreover, two studies [30,33] examined additional factors. One [30] analyzed pain and bacterial counts, whilst the second [33] analyzed pain, bacterial counts and radiographic healing. 

For the studies with incomplete parameter descriptions (5/17), the following deficiencies were found:

- Power: 1/5;
- Tip or spot size: 2/5;
- Fluence incorrectly calculated (consequently, either tip or energy was incorrect): 3/5;
- Pulse duration: 4/5;
- Energy per pulse: 1/5;
- Frequency: 1/5;
- Wet or dry canal: 1/5.

In addition to the above deficiencies, the spatial beam profile was not mentioned in any of the 17 studies examined.

Analysis of the correctly described protocols (12/17) has been performed for each laser application category as shown in Table 5, Table 6 and Table 7.

## 4. Discussion

Endodontic treatment conforming to the "state-of-the-art” demands the availability of sufficient chemo-mechanical instrumentation, with the adjunctive use of various irrigants. The most widely used and investigated of these are ethylenediamine tetra-acetate (EDTA) and sodium hypochlorite (NaOCl). The former is a chelating agent with no antibacterial effect per se, but which facilitates cleansing and the removal of infected tissue. However, the latter is a strong antimicrobial agent with the capacity to ‘dissolve’ the organic part of pulp residues and dentinal walls. It is used in various concentrations between 0.5 and 5.25% (w/v), a range in which a concentration higher than 2.5% (w/v) has not been clinically proven to be more effective [6].

Throughout the scientific literature, it is suggested that lasers cannot substitute such traditionally accepted endodontic therapies. The use of laser photonic energy within the root canal system of any tooth may be affected to a greater or lesser extent, by the consequences of thermal conduction, direct beam irradiation and the effects of refracted energy consequent to dentinal structure, root morphology, patency of access along the canal and the existence of multiple (accessory) root canals.

In vitro trials could not prove that the application of LAI with saline could efficiently replace NaOCl [4]. Specifically, De Meyer et al. showed that LAI applied by a 2940 nm laser system with saline could only reduce the viable counts of a dual-species biofilm by approximately 1 log_10_ unit, whereas LAI with NaOCl diminished these levels by >2.2 log_10_ units [4]. 

Similarly, Kreisler et al. showed that laser irradiation alone with an 809 nm diode laser in vitro was no more effective than the simultaneous use of the laser with NaOCl (1.49 log_10_ versus 2.84 log_10_ unit differences were observed, respectively). They concluded that the potential application of this diode laser should not be a substitute for conventional treatment, but should be regarded as a possible adjunctive treatment [45]. This was also supported by more recent studies [46]. Sohrabi et al. also noted that the use of a 980 nm laser system in a dry canal was significantly less effective than conventional chemomechanical treatment alone [47].

Both aPDT and PBM are essentially non-photothermal applications of laser photonic energy. The range of wavelengths for aPDT is currently 450–810 nm with a complementary application of photosensitizers. PBM effects in relation to this study may have direct application in pain modulation through the sub-ablative use of visible and near infra-red wavelengths, or may be an indirect benefit of surgical laser use at similar wavelengths within the canal and along a scatter gradient through the apex or dentinal tubules to the surrounding living tissue [12,24].

Chiniforush et al. outlined the fact that aPDT should be applied together with conventional chemo-mechanical techniques in order to further reduce the number of microorganisms, or alternatively modify their virulence factors, leading to a limited ability for them to form biofilms [12].

PBMT, in view of its sub-ablative action, clearly cannot replace a complete root canal treatment and hence this represents a purely adjunctive treatment modality.

Therefore, only clinical studies using lasers as an adjunct to the established, traditional methods were included in this systematic review.

Regarding the three different fields of laser application in non-surgical endodontic treatment explored in this review (conventional laser use, aPDT and PBMT), a tendency towards PBMT is clearly observable from the number of articles which could be included (i.e., 7/17).

With regard to the investigational objectives of the studies, pain, bacterial count and radiographic healing were examined. Specifically, conventional laser-assisted endodontic studies explored pain and bacteria, aPDT studies examined pain, bacteria and radiographic healing, and PBMT studies evaluated pain only.

It was also evident that most of the studies (14/17), independent of the fields considered (conventional, aPDT or PBMT), evaluated the effect of laser use in pain management as a major concern in the delivery of endodontic therapy. However, as a result of the subjective nature of pain perception, studies evaluating pain intensity were found to be highly heterogenous [19]. Consequently, in two of the studies [31,40], in addition to the visual analog scale (VAS)-pain evaluation, a quantification of calcitonin gene-related peptide (CRGP) in gingival crevicular fluid (GCF) and its correlation with pain, was assessed.

An interesting approach was taken by Arslan et al. in 2018 [40]. Using a 970 nm diode laser (with parameters listed in Table 3) with PBM as the second test group and a placebo laser system serving as a control, a sample size of 39 patients were tested for CGRP levels in GCF along with pain levels, after endodontic treatment and intracanal laser application in one of the groups [40]. CGRP is a pro-inflammatory mediator triggering neurogenic inflammation, during which pain sensitivity increases and pain threshold decreases [48]. These researchers concluded that conventional intra-canal laser application as well as PBM, exert an immunomodulatory effect. This was supported by the observation that in both laser treatment groups, the modification in CGRP levels for experimental teeth was closer to that of healthy contralateral teeth, than with endodontically-treated and contralateral teeth in the placebo group. Additionally, they were able to show a positive correlation of VAS scores of pain on percussion with both the pre- and post-operative total amount of CGRP in GCF [40].

Similarly, Yoo et al., using a 1440 nm Nd:YAG laser source (parameters available in Table 1), demonstrated that laser irradiation was significantly more effective in reducing pain on percussion (*p* = 0.003), and also in its ability to decrease substance P levels (*p* = 0.002), CGRP (*p*= 0.049), and MMP-8 (*p* = 0.002). VAS on percussion was positively correlated with substance P, CGRP, and MMP-8 concentrations [31]. 

It is plausible that for both studies, with protocols involving higher fluences being delivered, there was still a photobiomodulatory effect observed; although possibly lying outside the “biphasic dose response” range, this hypothesis is in agreement with Cronshaw et al., in that the dosimetry associated with pain relief applied in contemporary clinical practice lies within a higher range than that required for biostimulation [49].

Concerning the aPDT studies included in this review, de Miranda et al., found that using pain and bacterial count evaluations and radiographic healing criteria, they could show a significant difference in the periapical index score (PAI) at a six-month follow-up time [33]. This could be attributed to an improved healing axis with photodynamic therapy. This observation was primarily based on the antimicrobial action of this approach, and secondly on the ability of the laser photonic energy to effectively scatter and diffuse beyond the strict limits of the target tissue, hence photobiomodulating the area involved [50]. 

## 5. Conclusions

State-of-the-art, conventional endodontic therapy techniques continue to be the acknowledged as a “gold standard” treatment. Notwithstanding, the variety in reported failure rates and post-operative pain has prompted the requirement for adjunctive alternatives. In this context, the use of lasers has been thoroughly investigated. This systematic review aimed to explore the evidence of this technology’s clinical value. It was revealed that almost all studies (14/17) presented a statistically significant improved outcome in laser-assisted endodontic therapy. The remaining three did not show any differences over that of their corresponding control groups, but neither did they demonstrate any adverse effects. Therefore, lasers can be suggested as useful adjunctive treatment modalities.

As far as the safety of this treatment is concerned, a lack of parameter reporting, which especially in PBMT as well as in aPDT is of major importance, complicates a near-flawless conclusion. Irradiation protocols should be interpreted with special care regarding the thermal increase in the root canal system and that of the surrounding tissues. 

A tendency of research towards pain modulation in this field is developing. For future directions, more studies with clear and standardized protocols should be performed in order to further confirm the evidence base of this approach.

## Figures and Tables

**Table 1:** Conventional laser-assisted endodontics. Key: Tx = treatment, VAS = visual analog scale, rc = root canal.

Citation	Type of Study/Number of Samples	Test/Control Groups	Aim/ Approach	Laser/ Protocol	Follow-up	Outcome
**Genc Sen et al. (2019)** **[28]**	Parallel-group RCT/73 patients/single-rooted teeth retreatment	Laser + Conventional tx (37 patients)/ Conventional tx with placebo laser device (36 patients) 17% EDTA, and 2% NaOCl was used for the final irrigation in both groups.	Post-OP pain evaluation NRS 0–10 Percussion 0–2	940 nm 1 W 200 μm Tip starting from working length Speed of movement 2 mm/sec irradiation in circular motion 4 times each canal with 20 s intervals Canal DRY/ 1 session: day 0	3 days	Laser + RC tx group showed significant difference in VAS score (lower) after 24 h *p* = 0.036 and 72 h *p* = 0.016 Number of analgesic pills over 3 days significantly lower in laser group *p* = 0.008 Laser group Percussion on 4th day *p* = 0.008
**Dagher et al** **(2019)** **[29]**	Parallel-group RCT/56 patients/premolars and molars	PIPS Protocol + Conventional tx (25 patients)/ Conventional tx (31 patients) Both groups immediate obturation	Post-OP pain evaluation VAS daily Percussion day 7	2940 nm 20 mJ 50 μsec 15 Hz 0.3 W 600 μm radial stripped tip EDTA/ distilled water/NaOCl/distilled water 30 sec stationary irradiation in pulp chamber 30 s resting 4 cycles for NaOCl/ 1 session: day 0	Daily for 7 days	VAS: No difference between groups at any time Percussion: No difference between groups Pain in mastication: No difference between groups
**Morsy et al.** **(2018)** **[30]**	Parallel-group RCT/56 patients/maxillary central incisors with apical periodontitis	Laser + Conventional tx (28 patients)/ Conventional tx + placebo laser device (28 patients) Microbiological samples: 1.initial 2.after mechanical preparation 3.after laser day 0 (only Laser group) 4. day 7 (before irradiation) 5. day 7 (after irradiation for LG) before obturation 17% EDTA, and 2.5% NaOCl was used and Saline for the final irrigation	Pain (NRS) Micro-biology	980 nm 1.2 W gated mode 200 μm 1 mm from apex Speed of movement 2 mm/sec irradiation in helicoidal motion touching the canal walls 5 s each canal with 10-sec intervals 4 repetitions /2 sessions: day 0, day 7	7 days	Pain NRS: Laser group significantly better 6–12–24 h *p* < 0.001 48 h *p* = 0.002 7d *p* = 0.044 Bacteria: Laser group significantly better Both aerobic and anaerobic in all sampling times
**Yoo et al.** **(2013)** **[31]**	Parallel-group RCT/40 patients/teeth with persistent symptomatic apical periodontitis retreatment	Laser + Conventional tx (20 patients)/ Conventional tx (placebo laser) (20 patients) Root canal exudate to quantify the associated levels of substance P, calcitonin gene-related peptide (CGRP), and matrix metalloproteinase (MMP)-8 by immunoassay Both groups: copious 3.5% NaOCl was used as irrigation and filled with Ca(OH)_2_	Pain (VAS) Percussion (VAS) Reduction of pain-related neuropeptides and inflammatory cytokine levels in root canal exudates	1440 nm 0.2 W pulsed mode 1 Hz 200 mJ 300 μm tip apical 3 mm stationary (to prevent touching the walls) for 10 sec Canal DRY /1 session: day 0	3 days	Laser irradiation was significantly more effective in reducing pain on percussion (*p* = 0.003) and in decreasing substanceP (*p* = 0.002) CGRP (*p* = 0.049) and MMP-8 (*p* = 0.002) VAS-percussion was positively correlated with substanceP, CGRP, and MMP-8 levels. VAS-spontaneous pain was positively correlated with substanceP and MMP-8 levels. SubstanceP levels correlated directly with CGRP levels

**Table 2:** Antimicrobial photodynamic therapy (aPDT) in endodontic therapy.

Citation	Type of Study/Number of Samples	Test/Control Groups	Aim/ Approach	Laser + PS Used aPDT Protocol	Follow-up	Outcome
**Coelho et al.** **(2019)** **[32]**	Parallel-group RCT/60 patients/single-rooted teeth with fully developed apices, no probing and no mobility Rubber dam used	aPDT + Conventional rc tx (30 patients)/ Conventional rc tx (30 patients) Both groups received MB for 2 mins	Pain (VAS)	660 nm + MB (0.5 mg/mL) 2 mins incubation time 100 mW 180 s irradiation tip at working length in vertical motion 18 J 600 J/cm^2^ 1 session: day 0	7 days	aPDT + RC tx group showed significant difference in VAS score (lower) after 24 h and 72 h After 7 days no pain and no flare-up in both groups
**de Miranda et al. (2018)** **[33]**	Parallel-group RCT/16 patients/mandibular molars with apical periodontitis Rubber dam used	aPDT + Conventional rc tx (16 molars)/Conventional rc tx (16 molars) Both groups Ca(OH)_2_ for 7 days before obturation	Clinical symptoms (VAS) Periapical Index (PAI) Micro-biology	660 nm + MB (25 mg/mL) 5 mins incubation time 100 mW 300 s irradiation in vertical motion 300 μm tip 1 session: day 0	6 months	Clinically (VAS) no significant difference Microbiology no significant difference Radiographically significant better healing in aPDT group
**Pourhajibagher et al. (2017)** **[34]**	Repeated measures/14 patients with secondary-persistent endodontic infections (retreatments) Rubber dam used	Conventional endo re-treatment + aPDT Sampling before+after aPDT	Micro-biology	635 nm + TBO (0.025 mg/mL) 5 mins incubation time 220 mW 30 s irradiation 1 mm from WL 750 μm diffusor tip 1 session: day 0	Microbiological samples before/ after	Significant difference *p* < 0.05 in total bacteria count of secondary endodontic infection in aPDT group
**Juric et al. (2014)** **[35]**	Repeated measures/ 21 periapical periodontitis endo retreatment (endo ≥ 2 years) apical bone lesion 3 × 3 mm microbiological samples: 1. after access of canal 2. after endo re-treatment 3. after aPDT Rubber dam used	Conventional endo re-treatment + aPDT Sampling before+after aPDT	Micro-biology	660 + MB (10 mg/mL) 2 min incubation Wash with distilled water and dry 100 mW 60 s irradiation 450 μm diffusor tip 1 session: day 0	microbiological samples: 1. after access of canal 2. after endo re-treatment 3. after aPDT	chemomechanical preparation + aPDT vs. chemomechanical preparation alone significant difference in bacteria: gram positive (*p* = 0.02) gram negative (*p* = 0.005) facultative anaerobes (*p* = 0.013) obligate anaerobes (*p* = 0.007)
**Garcez et al. (2010)** **[36]**	Repeated measures/30 teeth of 21 patients periapical periodontitis, endo retreatment previously with antibiotic resistance apical bone lesion microbiological samples: 1. after access of canal 2. after endo re-treatment 3. after aPDT Rubber dam used	Conventional endo re-treatment + aPDT Sampling before+after aPDT Placing Ca(OH)_2_ for 7 days and then second aPDT session without sampling	Micro-biology	660 nm + polyethylenimine chlorin(e6) (3.6 mg/mL) 2 min incubation wash with distilled water and dry 40 mW 240 s irradiation 9.6 J 200 μm tip spiral movement 1 session: day 0	microbiological samples: 1. after access of canal 2. after endo re-treatment 3. after aPDT	The combination of endodontic therapy and aPDT killed all 9 multi-drug resistant bacterial species found in root canal infections no statistical analysis

**Table 3:** Photobiomodulation (PBM) in endodontic therapy.

Citation [ref]	Type of Study/Number of Samples	Test/Control Groups	Aim/ Approach	Laser/ Protocol	Follow-up	Outcome
**Nunes et al.** **(2019)** **[37]**	Parallel-group RCT/70 patients/mandibular molars with pulpitis	conventional rc tx + PBM (35 patients)/conventional rc tx + ibuprofen 600mg after 12 + 24h (35 patients)	Pain (VRS+NRS)	808 nm 100 mW CW Spot size 0.0283 cm^2^ 2 points buccal 2 points lingual corresponding to the apex of each root Contact with mucosa 25 s per point 2.5 J per point 90 J/cm^2^ per point Power meter 1 session: day 0	72 h	VRS: 6 h *p* < 0.001 12 h *p* = 0.005 24 h *p* = 0.001 72 h *p* = 0.317 (ns) NRS: 6 h *p* = 0.001 12 h *p* = 0.002 24 h *p* < 0.001 72 h *p* = 0.317 (ns)
**Lopes et al.** **(2019)** **[38]**	Parallel-group RCT/60 patients/mandibular molars with pulpitis	conventional rc tx + PBM (30 patients)/conventional rc tx (30 patients)	Pain (VRS + NRS) Possible reason of pain (regression analysis)	808 nm 100 mW CW Spot size 0.0283 cm^2^ 2 points buccal 2 points lingual corresponding to the apex of each root Contact with mucosa 25 s per point 2.5 J per point 90 J/cm^2^ per point 1 session: day 0	24 h	VRS: 6 h *p* = 0.123 12 h *p* = 0.127 24 h *p* = 0.013 NRS: 6 h *p* = 0.123 12 h *p* = 0.127 24 h *p* = 0.015 pain intensity associated with extrusion of root canal filling material-regression analysis
**Doganay et al. (2019)** **[39]**	Parallel-group RCT/26 patients/mandibular molars with symptomatic apical periodontitis	conventional rc tx + PBM (13 patients)/conventional rc tx + placebo (13 patients)	Pain (VAS) Substance P in gingival crevicular fluid (GCF) Immune-assay ELISA	970 nm 0.5 W 10 Hz Pulse width duty cycle 50% Tip-to-tissue 10 mm Apex area circular movement 200 μm tip Spot size 1.1569 cm^2^ 60 s per tooth 2.86 W/cm^2^ 1 session: day 0	7 days	Substance P Placebo group *p* = 0.553 PBM group significantly higher *p* = 0.005 VAS-percussion pain was significantly lower in PBM group Day 1 *p* = 0.006 Day 3 *p* = 0.019 Day 5 *p* = 0.011 Day 7 *p* = 0.046
**Arslan et al. (2018)** **[40]**	Parallel-group RCT/39 patients/mandibular molars with symptomatic apical periodontitis	conventional rc tx + PBM (13 patients)/conventional rc tx + intracanal laser (13 patients)/conventional rc tx + placebo (13 patients) GCF sample collected always also in contralateral tooth	Pain VAS-percussion (Pearson’s correlation) calcitonin gene-related peptide (CGRP) in the gingival crevicular fluid (GCF) GCF sample collected always also in contralateral tooth as control	970 nm Intracanal: 2 W, 200 μm tip WL-1 mm up-and-down motion under continuous irrigation with distilled water Irradiation time 60 s PBM: 0.5 W 10 Hz Tip-to-tissue 10 mm Apex area 200 μm tip 30 s per mesial and distal root 2.86 W/cm^2^ 1 session: day 0	VAS day 0 and 7 CGRP Day 0 and 7	CGRP: Placebo group significantly higher for experimental than control teeth Intracanal and PBM groups no significant difference between experimental and control teeth VAS: Pain on percussion positively correlated to total amount of CGRP
**Nabi et al. (2018)** **[41]**	Parallel-group RCT/120 patients/ teeth with pulpitis	conventional rc tx + PBM (30 patients)/ conventional rc tx + 400 ibuprofen 1 h before tx (30 patients)/ conventional rc tx + 400 ibuprofen 1 h before tx + PBM (30 patients)/ conventional rc tx (30 patients)	Pain Heft and Parker pain rating scale	905 nm 50 Hz 3 min irradiation buccal and lingual perpendicular to apex	48 h	24 h: PBM-only group vs. ibuprofen *p* = 0.04 (PBM less pain) PBM-only group vs. PBM+ibuprofen *p* = 0.455 (ns) PBM-only vs. no medication *p* = 0.004 (PBM less pain) 48h: PBM-only group vs. ibuprofen *p* = 0.046 (PBM less pain) PBM-only group vs. PBM+ibuprofen *p* = 0.808 (ns) PBM-only vs. no medication *p* = 0.002 (PBM less pain)
**Doganay et al.** **(2018)** **[42]**	Parallel-group RCT/42 patients mandibular molars with symptomatic apical periodontitis	conventional rc tx + PBM (14 patients)/ conventional rc tx + placebo (14 patients)/ Conventional only rc tx (14 patients)	Pain (VAS) Percussion-pain (VAS)	970 nm 0.5 W 10 Hz Tip-to-tissue 10 mm Apex area 8 mm tip 30 s per mesial and distal root 2.86 W/cm^2/^ 1 session: day 0	7 days	PBM-group lower pain *p* < 0.05 in day 1 and 3 Percussion at day 7 no significant difference
**Arslan et al.** **(2017)** **[43]**	Parallel-group RCT/36 patients mandibular molars with periapical lesion	conventional rc tx + PBM (18 patients)/ conventional rc tx (18 patients)	Pain (VAS) and number of analgesics Percussion	970 nm 0.5 W 10 Hz Tip-to-tissue 10 mm Apex area 8 mm tip 30 s per mesial and distal root 2.86 W/cm^2^/ 1 session: day 0	7 days	PBM-group lower pain *p* < 0.05 in first four days Number of analgesics taken significantly lower Percussion day 7 no significant difference
**Asnaashari et al.** **(2017)** **[44]**	Parallel-group RCT/61 patients Retreatment of maxillar and mandibular molars	conventional rc tx + PBM (41 patients)/ conventional rc tx (20 patients)		808 nm 100 mW 70 J/cm^2^ 80 s 600 μm tip Buccal and lingual apical area/ 1 session: day 0		VAS and analgesic consumption not statistically significant at any time

**Table 4:** Risk of bias assessment.

Citation [ref]	Randomization	Sample Size Calculation and Required Number Included	Allocation Ratio of 1:1	Baseline Situation Similar	Blinding	Parameters of Laser Use Described Appropriately and Calculations Correct	Power Meter Used	Numerical Results Available (Statistics)	Outcome Data Complete	Correct Inter-pretation of Data	Total Score/10
**Endo + ConvLas**											
Genc Sen et al. (2019) [28]	yes	yes	yes	yes	yes	yes	no	yes	yes	yes	9
Dagher et al. (2019) [29]	yes	no	no	yes	yes	yes	no	yes	yes	yes	7
Morsy et al. (2018) [30]	yes	yes	yes	yes	yes	no	no	yes	yes	yes	9
Yoo et al. (2013) [31]	yes	yes	yes	yes	yes	no	no	yes	yes	yes	9
**Endo + aPDT**											
Coelho et al. (2019) [32]	yes	yes	yes	yes	yes	yes	no	yes	yes	yes	9
de Miranda et al. (2018) [33]	yes	yes	yes	yes	yes	yes	no	yes	yes	yes	9
Pourhajibagher et al. (2017) [34]	yes	no	yes	yes	yes	yes	no	yes	yes	yes	8
Juric et al (2014) [35]	yes	no	yes	yes	yes	yes	no	yes	yes	yes	8
Garcez et al. (2010) [36]	yes	no	yes	yes	yes	yes	no	no	yes	yes	7
**Endo + PBM**											
Nunes et al. (2019) [37]	yes	yes	yes	yes	yes	yes	yes	yes	yes	yes	10
Lopes et al. (2019) [38]	yes	yes	yes	yes	yes	yes	no	yes	yes	yes	9
Doganay et al. (2019) [39]	yes	no	yes	yes	yes	no	no	yes	yes	yes	7
Arslan et al. (2018) [40]	yes	no	yes	yes	yes	yes	no	yes	yes	yes	8
Nabi et al. (2018) [41]	yes	no	yes	yes	no	no	no	yes	yes	yes	6
Doganay et al. (2018) [42]	yes	no	yes	yes	yes	yes	no	yes	yes	yes	8
Arslan et al. (2017) [43]	yes	yes	yes	yes	yes	yes	no	yes	yes	yes	9
Asnaashari et al. (2017) [44]	yes	yes	no	yes	no	no	no	yes	yes	yes	6

**Table 5:** Parameters used in conventional laser endodontics.

Conventional	1 Study [28]	1 Study [29]
Wavelength (nm)	940	2940
Power (W)	1	0.3
Energy per pulse (mJ)	CW	20
Pulse duration (μs)	CW	50
Frequency (Hz)	CW	15
Tip (μm)	200	600
Tip localization	Working length	Pulp chamber
Speed of movement (mm/s)	2	0
Kind of motion	Circular	None
Irradiation time (s)	Depending on root canal length	30
Time-intervals (s)	20	30
Repetition of irradiation cycles	4	4
Wet or Dry canal	Dry	EDTA/water/ NaOCl/water
Number of sessions	1	1

**Table 6:** Parameters used in antimicrobial photodynamic therapy (aPDT) in endodontics.

aPDT	1 Study [36]	1 Study [34]	3 Studies [32,33,35]		
Combination PS + Laser Wavelength	PEI-ce6 + 660 nm	TBO + 635 nm	MB + 660 nm		
PS-concentration (mg/mL)	3.6	0.025	0.5	25	10
Incubation time (min)	2	5	2	5	2
Power (mW)	40	220	100	100	100
Irradiation time (s)	240	30	180	300	60
Tip (μm)	200	750	200	300	450 diffusor
Number of sessions	1	1	1	1	1

**Table 7:** Parameters used in PBM in endodontics.

PBM	2 Studies [37,38]	1 Study [40]	2 Studies [42,43]
Wavelength (nm)	808	970	970
Power (W)	0.1	0.5	0.5
Energy per pulse (mJ)	CW	25	25
Pulse duration (ms)	CW	50	50
Frequency (Hz)	CW	10	10
Tip (μm)	1900	200	8000
Tip-to-tissue distance (mm)	0	10	10
Speed of movement (mm/s)	0	0	0
Irradiation time (s)	25 per point	30 per root	30 per root
Fluence (J/cm^2^)	90 per point	43.1 per root	6.1 per root
Number of sessions	1	1	1

## References

[1] Ogle O.E. (2017). Odontogenic Infections. Dent. Clin. N. Am..

[2] Neelakantan P., Romero M., Vera J., Daood U., Khan A.U., Yan A., Cheung G.S.P. (2017). Biofilms in Endodontics-Current Status and Future Directions. Int. J. Mol. Sci..

[3] Peters L., Wesselink P., Buijs J., VanWinkelhoff A. (2001). Viable Bacteria in Root Dentinal Tubules of Teeth with Apical Periodontitis. J. Endod..

[4] De Meyer S., Meire M.A., Coenye T., De Moor R.J.G. (2017). Effect of laser-activated irrigation on biofilms in artificial root canals. Int. Endod. J..

[5] Berutti E., Marini R., Angeretti A. (1997). Penetration ability of different irrigants into dentinal tubules. J. Endod..

[6] Haapasalo M., Udnaes T., Endal U. (2003). Persistent, recurrent, and acquired infection of the root canal system post-treatment. Endod. Top..

[7] Moreira M.S., Anuar A.S.N.S., Tedesco T.K., dos Santos M., Morimoto S. (2017). Endodontic Treatment in Single and Multiple Visits: An Overview of Systematic Reviews. J. Endod..

[8] Riis A., Taschieri S., Del Fabbro M., Kvist T. (2018). Tooth Survival after Surgical or Nonsurgical Endodontic Retreatment: Long-term Follow-up of a Randomized Clinical Trial. J. Endod..

[9] Teo C.Y.J., George R., Walsh L.J. (2018). Dispersion of near-infrared laser energy through radicular dentine when using plain or conical tips. Lasers Med. Sci..

[10] Kasić S., Knezović M., Beader N., Gabrić D., Malčić A.I., Baraba A. (2017). Efficacy of Three Different Lasers on Eradication of Enterococcus faecalis and Candida albicans Biofilms in Root Canal System. Photomed. Laser Surg..

[11] Granevik Lindström M., Wolf E., Fransson H. (2017). The Antibacterial Effect of Nd:YAG Laser Treatment of Teeth with Apical Periodontitis: A Randomized Controlled Trial. J. Endod..

[12] Chiniforush N., Pourhajibagher M., Shahabi S., Kosarieh E., Bahador A. (2016). Can antimicrobial photodynamic therapy (aPDT) enhance the endodontic treatment?. J. Lasers Med. Sci..

[13] Macedo R.G., Wesselink P.R., Zaccheo F., Fanali D., Van Der Sluis L.W.M. (2010). Reaction rate of NaOCl in contact with bovine dentine: Effect of activation, exposure time, concentration and pH. Int. Endod. J..

[14] Garcia-Diaz M., Huang Y.Y., Hamblin M.R. (2016). Use of fluorescent probes for ROS to tease apart Type I and Type II photochemical pathways in photodynamic therapy. Methods.

[15] Diogo P., Faustino M.F.A., Neves G.M.P.M.S., Palma P.J., Baptista I.P., Gonçalves T., Santos J.M. (2019). An insight into advanced approaches for photosensitizer optimization in endodontics—A critical review. J. Funct. Biomater..

[16] Nagendrababu V., Pulikkotil S.J., Jinatongthai P., Veettil S.K., Teerawattanapong N., Gutmann J.L. (2019). Efficacy and Safety of Oral Premedication on Pain after Nonsurgical Root Canal Treatment: A Systematic Review and Network Meta-analysis of Randomized Controlled Trials. J. Endod..

[17] Comparin D., Moreira E., Souza E., De-Deus G., Arias A., Silva E. (2017). Postoperative Pain after Endodontic Retreatment Using Rotary or Reciprocating Instruments: A Randomized Clinical Trial. J. Endod..

[18] Shamszadeh S., Shirvani A., Eghbal M.J., Asgary S. (2018). Efficacy of Corticosteroids on Postoperative Endodontic Pain: A Systematic Review and Meta-analysis. J. Endod..

[19] Chen Y., Chen X.L., Zou X.L., Chen S.Z., Zou J., Wang Y. (2019). Efficacy of low-level laser therapy in pain management after root canal treatment or retreatment: A systematic review. Lasers Med. Sci..

[20] Alí A., Olivieri J.G., Duran-Sindreu F., Abella F., Roig M., García-Font M. (2016). Influence of preoperative pain intensity on postoperative pain after root canal treatment: A prospective clinical study. J. Dent..

[21] Chow R.T., Armati P.J., Laakso E.L., Bjordal J.M., Baxter G.D. (2011). Inhibitory effects of laser irradiation on peripheral mammalian nerves and relevance to analgesic effects: A systematic review. Photomed. Laser Surg..

[22] Vahdatinia F., Gholami L., Karkehabadi H., Fekrazad R. (2019). Photobiomodulation in Endodontic, Restorative, and Prosthetic Dentistry: A Review of the Literature. Photobiomodulation, Photomed. Laser Surg..

[23] Chow R.T., Armati P.J. (2016). Photobiomodulation: Implications for anesthesia and pain relief. Photomed. Laser Surg..

[24] De Freitas L.F., Hamblin M.R. (2016). Proposed Mechanisms of Photobiomodulation or Low-Level Light Therapy. IEEE J. Sel. Top. Quantum Electron..

[25] Parker S., Coluzzi D., Parker S. (2017). Laser-tissue interaction and photobiomodulation Chapter 3.14. Lasers in Dentistry-Current Concepts.

[26] Moher D., Liberati A., Tetzlaff J., Altman D.G., Group T.P. (2009). Preferred Reporting Items for Systematic Reviews and Meta-Analyses: The PRISMA Statement (Reprinted from Annals of Internal Medicine). PLOS Med..

[27] Higgins J., Savović J., Page M., Elbers R., Sterne J., Higgins J., Thomas J., Chandler J., Cumpston M., Li T., Page M., Welch V. (2019). Assessing risk of bias in a randomized trial. Cochrane Handbook for Systematic Reviews of Interventions.

[28] Genc Sen O., Kaya M. (2019). Effect of root canal disinfection with a diode laser on postoperative pain after endodontic retreatment. Photobiomodulation Photomed. Laser Surg..

[29] Dagher J., El Feghali R., Parker S., Benedicenti S., Zogheib C. (2019). Postoperative Quality of Life Following Conventional Endodontic Intracanal Irrigation Compared with Laser-Activated Irrigation: A Randomized Clinical Study. Photobiomodulation Photomed. Laser Surg..

[30] Morsy D.A., Negm M., Diab A., Ahmed G. (2018). Postoperative pain and antibacterial effect of 980 nm diode laser versus conventional endodontic treatment in necrotic teeth with chronic periapical lesions: A randomized control trial. F1000 Res..

[31] Yoo Y.J., Shon W.J., Baek S.H., Kang M.K., Kim H.C., Lee W. (2014). Effect of 1440-nanometer neodymium:Yttrium-aluminum-garnet laser irradiation on pain and neuropeptide reduction: A randomized prospective clinical trial. J. Endod..

[32] Coelho M.S., Vilas-Boas L., Tawil P.Z. (2019). The effects of photodynamic therapy on postoperative pain in teeth with necrotic pulps. Photodiagnosis Photodyn. Ther..

[33] de Miranda R.G., Colombo A.P.V. (2018). Clinical and microbiological effectiveness of photodynamic therapy on primary endodontic infections: A 6-month randomized clinical trial. Clin. Oral Investig..

[34] Pourhajibagher M., Ghorbanzadeh R., Parker S., Chiniforush N., Bahador A. (2017). The evaluation of cultivable microbiota profile in patients with secondary endodontic infection before and after photo-activated disinfection. Photodiagnosis Photodyn. Ther..

[35] Jurič I.B., Plečko V., Pandurić D.G., Anić I. (2014). The antimicrobial effectiveness of photodynamic therapy used as an addition to the conventional endodontic re-treatment: A clinical study. Photodiagnosis Photodyn. Ther..

[36] Garcez A.S., Nuñez S.C., Hamblin M.R., Suzuki H., Ribeiro M.S. (2010). Photodynamic therapy associated with conventional endodontic treatment in patients with antibiotic-resistant microflora: A preliminary report. J. Endod..

[37] Nunes E.C., Herkrath F.J., Suzuki E.H., Gualberto Júnior E.C., Marques A.A.F., Sponchiado Júnior E.C. (2019). Comparison of the effect of photobiomodulation therapy and Ibuprofen on postoperative pain after endodontic treatment: Randomized, controlled, clinical study. Lasers Med. Sci..

[38] Lopes Barros L., Herkrath F.J. (2019). Effect of photobiomodulation therapy on postoperative pain after endodontic treatment: A randomized, controlled, clinical study. Clin. Oral Investig..

[39] Doğanay Yildiz E., Arslan H., Köseoğlu S., Arabaci T., Yildiz D.A., Savran L. (2019). The effect of photobiomodulation on total amount of substance P in gingival crevicular fluid: Placebo-controlled randomized clinical trial. Lasers Med. Sci..

[40] Arslan H., Köseoğlu S., Doğanay Yildiz E., Arabaci T., Savran L., Yildiz D.A., Veyisoğlu G. (2018). Effect of intracanal diode laser application and low-level laser therapy on CGRP change. Braz. Oral Res..

[41] Nabi S., Amin K., Masoodi A., Farooq R., Purra A.R., Ahangar F.A. (2018). Effect of preoperative ibuprofen in controlling post endodontic pain with and without low-level laser therapy in single visit endodontics: A randomized clinical study. Indian J. Dent. Res..

[42] Doğanay Yildiz E., Arslan H. (2018). Effect of Low-level Laser Therapy on Postoperative Pain in Molars with Symptomatic Apical Periodontitis: A Randomized Placebo-controlled Clinical Trial. J. Endod..

[43] Arslan H., Doğanay E., Karataş E., Ünlü M.A., Ahmed H.M.A. (2017). Effect of Low-level Laser Therapy on Postoperative Pain after Root Canal Retreatment: A Preliminary Placebo-controlled, Triple-blind, Randomized Clinical Trial. J. Endod..

[44] Asnaashari M., Ashraf H., Daghayeghi A.H., Mojahedi S.M., Azari-Marhabi S. (2017). Management of post endodontic retreatment pain with low level laser therapy. J. Lasers Med. Sci..

[45] Kreisler M., Kohnen W., Beck M., Al Haj H., Christoffers A., Götz H., Duschner H., Jansen B. (2003). Efficacy of NaOCl/H_2_O_2_ Irrigation and GaAlAs Laser in Decontamination of Root Canals In Vitro. Lasers Surg. Med..

[46] Sarda R.A., Shetty R.M., Tamrakar A., Shetty S.Y. (2019). Antimicrobial efficacy of photodynamic therapy, diode laser, and sodium hypochlorite and their combinations on endodontic pathogens. Photodiagnosis Photodyn. Ther..

[47] Sohrabi K., Sooratgar A., Zolfagharnasab K., Kharazifard M.J., Afkhami F. (2016). Antibacterial activity of diode laser and sodium hypochlorite in enterococcus faecalis-contaminated root canals. Iran. Endod. J..

[48] Rechenberg D.K., Galicia J.C., Peters O.A. (2016). Biological markers for pulpal inflammation: A systematic review. PLoS ONE.

[49] Cronshaw M., Parker S., Arany P. (2019). Feeling the Heat: Evolutionary and Microbial Basis for the Analgesic Mechanisms of Photobiomodulation Therapy. Photobiomodulation Photomed. Laser Surg..

[50] Wainwright M., Maisch T., Nonell S., Plaetzer K., Almeida A., Tegos G.P., Hamblin M.R. (2017). Photoantimicrobials-are we afraid of the light?. Lancet Infect. Dis..

